# Retraction Note to: High systemic levels of the cytokine-inducing HMGB1 isoform secreted in severe macrophage activation syndrome

**DOI:** 10.1186/s10020-020-00263-2

**Published:** 2020-12-30

**Authors:** Karin Palmblad, Hanna Schierbeck, Erik Sundberg, Anna-Carin Horne, Helena Erlandsson Harris, Jan-Inge Henter, Daniel J. Antoine, Ulf Andersson

**Affiliations:** 1 Unit of Pediatric Rheumatology, Department of Women’s and Children’s Health, Karolinska Institutet, Karolinska University Hospital, Solna, 171 76 Stockholm, Sweden; 2 Rheumatology Unit, Department of Medicine, Karolinska Institutet, Karolinska University Hospital, Solna, Stockholm, Sweden; 3 Childhood Cancer Research Unit, Department of Women’s and Children’s Health, Karolinska Institutet, Karolinska University Hospital, Solna, Stockholm, Sweden; 4 Medical Research Council Centre for Drug Safety Science, Department of Molecular and Clinical Pharmacology, University of Liverpool, Liverpool, UK

## Retraction Note to: Mol Med (2014) 20:538–547 10.2119/molmed.2014.00183

The Editors-in-Chief have retracted this article (Palmblad et al. 2014) following an investigation by the University of Liverpool. The investigation concluded, based on the circumstances described below, that the data obtained by mass-spectrometry presented in Figs. 2–5 are likely to be fraudulent and should not be relied upon. The investigation found no evidence that this mass-spectrometry was carried out on any University of Liverpool instrumentation from systematic examination of equipment logs. This does not exclude the fact that the mass-spectrometry may have been undertaken at another research center. However, they found no evidence that the mass-spectrometry was performed anywhere else or any financial audit trail to indicate that instrument time was paid for elsewhere. In addition to this, the mass-spectrometry technique to quantify HMGB-1 isoforms by mass-spectrometry has been unable to be reproduced at the Liverpool laboratories using either the supplied protocol or through adaptation of the protocols. Similar data in other publications investigated in this case, in which the institution has been able to assess primary data or which involve publication of assessable mass-spectra images, have demonstrated figure fabrication and fraudulent manipulation of data. The co-authors of the article were found by the investigation not to be complicit in any research misconduct, and they have been invited to resubmit a revised version of the manuscript for further peer review. More information on the university's investigation can be found on the university website (Further update on research misconduct investigation 2020).

Dr. Karin Palmblad, Dr. Anna-Carin Horne, Dr. Helena Erlandsson Harris, Dr. Jan-Inge Henter and Dr. Ulf Andersson agree to this retraction. Dr. Hanna Schierbeck and Dr. Erik Sundberg have not responded to any correspondence from the editor/publisher about this retraction.
